# DVWA gene polymorphisms and osteoarthritis

**DOI:** 10.1186/s13104-015-0987-1

**Published:** 2015-02-04

**Authors:** Valentina Bravatà, Luigi Minafra, Giusi I Forte, Francesco P Cammarata, Michele Saporito, Filippo Boniforti, Domenico Lio, Maria C Gilardi, Cristina Messa

**Affiliations:** IBFM CNR-LATO, Cefalù, PA Italy; Clinica Ortopedica e Traumatologica, Università degli Studi di Palermo, Palermo, Italy; Unità Operativa di Ortopedia, San Raffaele Hospital “G. Giglio”, Cefalù, PA Italy; Department of Pathobiology and Medical and Forensic Biotechnologies, University of Palermo, Palermo, Italy; Nuclear Medicine, San Raffaele Scientific Institute, Milan, Italy; Department of Health Sciences, Tecnomed Foundation, University of Milano-Bicocca, Milan, Italy; Nuclear Medicine Center, San Gerardo Hospital, Monza, Italy

**Keywords:** Osteoarthritis, DVWA, Single nucleotide polymorphisms, Haplotypes, KL

## Abstract

**Background:**

Osteoarthritis (OA) is a degenerative joints disorder influenced by genetic predisposition. We reported that rs11718863 DVWA SNP was represented in Sicilian with a more severe Kellgren and Lawrence (KL) radiographic grade, displaying its predictive role as OA marker progression. Here, we describe the DVWA SNPs: rs11718863, rs7639618, rs7651842, rs7639807 and rs17040821 probably able to induce protein functional changes.

**Findings:**

Sixty-one Sicilian patients with knee OA and 100 healthy subjects were enrolled. Clinical and radiographic evaluation was performed using AKSS scores and KL. Linkage Disequilibrium (LD) analyses were performed in order to verify whether the SNPs segregate as haplotype. All DVWA SNPs’MinorAllele Frequencies (MAF) were greater than in the European. The rs7639618 SNP showed a statistical association with KL. Our analyses show that a LD exists among rs11718863 and rs7639618, as well as between rs7651842, rs7639807 and rs17040821 SNPs. We also observed that three out of the 161 individuals investigated were simultaneously homozygous carriers of the rs7651842, rs7639807 and rs17040821 MAF alleles.

**Conclusions:**

In summary, the purpose of this preliminary research was to highlight possible associations between DVWA SNPs and OA clinical and radiographic data. This work represents a multidisciplinary medicine approach to study OA where clinical, radiological and genetic evaluation could contribute to better define OA grading.

## Background

Osteoarthritis (OA) is a multifactorial, inflammatory and degenerative joints disorder characterized by degeneration of articular cartilage, intra-articular inflammation with synovitis, and changes in peri-articular and subchondral bone [1]. OA may be considered in the category of polygenic diseases [2-4]. OA diagnosis is mainly clinical and radiological and X-ray images are the gold standard to confirm the clinical diagnosis and to grade the disease [5-7]. Many studies analyzed the correlation between knee OA radiographic data and clinical status of the affected joint by using specific clinical scores and radiographic scales. In this context, the Kellgren and Lawrence radiographic grading scale (KL) of knee OA, has been shown to be accurate in OA classification by several authors [8-12].

Nowadays, molecular genetic investigations have gained an increasingly significant role in the knowledge of OA etiology and have provided evidence for a genetic component to OA [13-15]. Thanks to recent genome wide association (GWA) studies, it is known that susceptibility to OA is influenced by genetic predisposition. Searching for OA susceptibility loci, it has become apparent that many of these have particular relevance for the disease development at particular skeletal sites and furthermore, some loci could be linked to the disease, depending on OA patients ethnic differences [8,16-18].

On the Online Mendelian Inheritance in Man database (OMIM), which collects human genes and genetic disorders with particular focus on the molecular relationship between genetic variation and phenotypic expression, the following polymorphisms and aspartic acid (D) repeats are reported as OA susceptibility (OS) sites: FRZB rs288326 (OS1A) and rs7775 (OS1B), MATN3 rs77245812 (OS2), ASPN D14 repeats (OS3), PTHR2 rs76758470 (OS4), GDF5 rs143383 (OS5) and DVWA rs11718863 (OS6) [19].

Moreover, several associations studies between SNPs and OA disease remain unconfirmed or controversial, due to bias in patient enrolling criteria differences in OA affected joint sites, in classification and staging modes.

In turn, to our knowledge, genetic contribution to KL radiographic severity in knee OA was previously described only by our group in Sicilian individuals and by Valdes et colleagues in UK OA patients and no information are available on people from other Europe countries [20,21].

Considering that polymorphisms are involved in biodiversity and could be affected by ethnic heritage and geographic localization, it becomes of interest to explore the geographical and ethnic allele distribution, extremely important in fully understanding the SNP variants effects. More precisely, Sicilian individuals have a specific genetic background and different alleles distribution compared to the rest of Europe (and also compared to the rest of Italy), probably due to the ethnical, geographic and distinct gene–environment interactions certainly caused by deep human migration movements, occurred in Sicily across centuries as described by several authors [22-25].

Recently, we described that OS5 and OS6 were statistically associated with clinical features such as KL radiographic grading scale in Sicilian OA patients, thus contributing in better define OA grading and progression.

Particularly, we observed that in our cohort of patients, OS6 genetic alteration (DVWA rs11718863 SNP) was more represented in patients with more severe OA KL radiographic grading with poor joint function, displaying its predictive role as OA marker progression [20].

DVWA gene (gene ID: 344875), also known as COL6A4P1 (collagen type VI, alpha 4 pseudogene 1), specifically expressed in cartilage, encodes for a protein showing double von Willebrand factor A domains (VWA domain), involved in cellular adhesion and protein-to-protein interactions [20]. DVWA rs11718863 and rs7639618 polymorphisms are localized in the same exonic region (the third) and cause missense mutations with a consequent amino acidic substitution (Tyr169Asn and Cys260Tyr, respectively). These SNPs were described as involved in the decrease of the strength interaction between the DVWA protein and the β-tubulin [26-28], this protein-protein binding is important in the regulation of chondrocyte differentiation, which protects articulated joints from OA onset.

Several DVWA gene functional variants were assayed by Miyamoto et colleagues in a large GWA association study. In particular, DVWA rs11718863 SNP is reported to be strongly associated with risk of knee OA (OR = 1.43 *p* < 7x10-11) and able to influence β-tubulin binding in Asian populations [29].

In addition, both Valdes et al. and Meulenbelt et al showed an association between this genetic alteration and knee OA in UK population not confirmed for other European countries. However, the higher risk allele frequency in the European samples highlighted the ethnic different penetrance of OA susceptibility genes and, once again, the need to evaluate the alleles geographic distribution [26].

Interestingly, as suggested by Miyamoto et al. both rs11718863 DVWA and rs7639618 polymorphisms could have a role in etiopathogenesis of OA in Japanese and Chinese populations. Furthermore, the Tyr169-Cys260 double mutated protein isoform, could have a weaker binding capacity to β-tubulin and seems to be over-represented in OA, further suggesting that this interaction might be fundamental to protect joints from OA [29].

Based on these considerations, in the present work we wanted to test the association of these two regulative SNPs with OA disease in our Sicilian cohort and we evaluated their association with KL grading scale.

To our knowledge, the association between KL and rs7639618 DVWA SNP has been assayed for the first time, in this work.

Moreover, even if Valdes et al and Meulenbelt et al analyzed the above-mentioned DVWA SNPs in knee OA cases, only Miyamoto and colleagues suggested that rs11718863 and rs7639618 DVWA SNPs might act as haplotype rather than single SNPs in Japanese and Chinese OA cohorts [18,26,29]. So, additional investigations are needed in order to evaluate their role in OA care, according to the patients ethnic origin.

Recently, Meulenbelt et al conducted a large replication study and a meta-analyses of DVWA polymorphisms, showing that the minor allele frequencies (MAFs) for these SNPs are much lower in the European individuals than in Asian ones, even if the Japanese and Chinese populations are much more investigated. Additionally, different results are reported within the European studies, probably due to selection criteria, different clinical evaluations, control group selection and geographical localizations, underlying the need of additional standardization of the OA phenotypes [21,26-29].

Furthermore, despite the potential functional influence of these two SNPs on OA etiology and pathogenesis, the number of scientific papers on this topic is still limited [4,21,26-38]. Then, it becomes of interest to explore geographical and ethnic distribution of the polymorphic alleles considered extremely important for a full understanding of OA susceptibility risk and to develop proper treatments related to the individual genetic background.

## Patient and methods

### Patients

On admission to hospital, sixty-one Sicilian patients, affected with knee primary OA, aged 54-86 years old and candidates for knee arthroscopy or arthroplasty, were enrolled in this project. We also enrolled 100 healthy Sicilian subjects as control samples. This study (named OA_BIOMOL_1) was approved by the Ethical Committee of the San Raffaele G. Giglio Hospital, Cefalù, Italy (number of protocol: CE 2011/63) and the patients gave their written informed consent according to the Helsinki Declaration.

### Clinical and radiographic evaluation

Clinical evaluation was performed for each patient before surgery using the American Knee Society Score (AKSS), that includes two sub-scores: Knee Score (KS) and Function Score (FS) as described previously [20]. The radiographic evaluation was performed on knee antero-posterior and lateral X-ray views by a single investigator, using the KL grading scale which includes 4 grades: grade 1 – possible narrowing of joint space (NJS) and possible presence of osteophytes; grade 2 – definite NJS and definite osteophytes; grade 3 – definite NJS, multiple osteophytes, sclerosis, cysts and possible deformity of bone contour; grade 4 – marked NJS, large osteophytes, severe sclerosis, cysts and definite deformity of bone contour [39,40]. The evaluation was undertaken on X-ray performed no more than four months before surgery. In our study we grouped grade 1 and 2 into a single grade because the radiographic differences in our cohort were considered not relevant compared to the ones between KL grade 3 and 4. So, we summarized the KL classification into 3 groups: A (grade 1 and 2), B (grade 3), C (grade 4).

### Genetic analysis

The patients were genotyped by sequencing analysis for rs11718863, rs7639618, rs7651842, rs7639807 and rs17040821 DVWA genetic polymorphisms. Human Gene Mutation Database (http://www.hgmd.cf.ac.uk) and dbSNP Short Genetic Variations database (http://www.ncbi.nlm.nih.gov/snp) were used to analyze gene regions containing the selected SNPs. Genomic DNA was extracted from peripheral blood using QIAamp DNA blood mini kit, according to the manufacturer’s specifications (Qiagen). After quality and quantity analysis, DNA was PCR amplified using primers designed by the Primer3 software (http://fokker.wi.mit.edu/primer3). Primer sequences used for the abovementioned DVWA SNPs detection were: 5’-aggctgcctgccattattctt-3’ and 5’-cccatgctgtttcctttgaaca -3’for forward and reverse primers respectively. PCR reactions were performed with 50 ng of genomic DNA in a total volume of 50 μL containing 1X PCR Gold Buffer, 1.5 mM di MgCl2, 200 μM dNTPs, 200 nM of forward and reverse primer mix, 1.25 U of AmpliTaq Gold DNA Polymerase (Applied Biosystems). The thermal cycle profile employed a 5-min denaturing step at 94°C, followed by 35 cycles at 94°C for 45 sec, 59°C for 45 sec, 72°C for 45 sec and a final extension step of 5 min at 72°C. Quality and quantity of PCR products were assessed on Bioanalyzer instrument (Agilent Technologies) and were purified using QIAquick PCR purification kit, according to the manufacturer’s specifications (Qiagen). To perform DNA sequencing, purified amplicons (924 bp) were labelled with BigDye Terminator v3.1 Cycle Sequencing Kit following the manufacturer’s standard protocol (Applied Biosystems). The thermal cycle profile employed 1 min denaturing step at 96°C, followed by 25 cycles at 96°C for 10 sec, 54°C for 5 sec, 60°C for 3 min. Labelled samples were purified with X-terminator purification kit according to manufacturer’s standard protocol and loaded in 3500-Dx Genetic Analyzer (Applied Biosystems) for separation by capillary electrophoresis. Electropherograms and sequence files were analyzed using Sequencing Analysis and SeqScape softwares (Applied Biosystems).

### Statistical analysis

The association between DVWA SNPs genotypes and KL groups, was analyzed using GraphPad InStat software version 3.05 (San Diego California USA) as previously described [20,25]. Mann Whitney-U test, Pearson’s Chi-Square test and Fisher’s exact test were performed. Differences in groups were considered significant when the *p-value* was less than or equal to 0.05. Hardy-Weinberg Equilibrium (HWE) was calculated. Finally, in order to verify the degree of allelic segregation among the SNPs of our interest, we calculated the Linkage Disequilibrium (LD) coefficients (D’ and r^2^) using Haploview software 3.32.

## Findings

### Clinical and radiographic evaluation

We recruited 61 cases, 36 females and 25 males, and they were divided into 3 groups (A, B, C) depending on the degree of radiographic knee OA.

According to the clinical scores we classified the patients as follows:

- Group A consisted of 20 patients, 11 females and 9 males,
- Group B consisted of 21 patients, 15 females and 6 males,
- Group C consisted of 20 patients, 10 females and 10 males.

### Linkage disequilibrium analysis

Firstly, in order to verify the degree of allelic segregation among the SNPs of our interest, we calculated the Linkage Disequilibrium (LD) coefficients (D’ and r^2^) using Haploview software 3.32. Based on the HapMap project databases [41-43], this approach was used for rs7639618, rs7639807, rs7651842 by a pairwise tagging mode, because allelic frequencies from different populations are available on this platform for these three SNPs. Instead, no data were available for the other two SNPs, rs17040821 and rs11718863.

Then, we replicated the D’ and r^2^ calculation for all the five above-mentioned SNPs, by using the allelic frequencies from our Sicilian cohort of healthy subjects, with the proper formulas: D’ = D/Dmax, and r^2^ = D2/p^1^p^2^q1q^2^ where, D = (x11)(x22) – (x12)(x21); Dmax is the smaller of p1q2 and p2q1, where the haplotype frequencies for the hypothetic loci A and B are defined as described in the Table 1. For our Sicilian cohort, the pairwise LDs for rs7639618, rs7639807 and rs7651842 perfectly matched with those observed by Haploview software (Table 1).

**Table 1:** **Haplotype and allele frequencies used for Linkage Disequilibrium analyses**

**Haplotype**	**Frequency**	**Allele**	**Frequency**
*A1B1*	*x*_*11*_	*A* **1**	*p*_*1*_ *= x*_*11*_ *+ x*_*12*_
*A1B2*	*x*_*12*_	*A* **2**	*p*_*2*_ *= x*_*21*_ *+ x*_*22*_
*A2B1*	*x*_*21*_	*B* **1**	*q*_*1*_ *= x*_*11*_ *+ x*_*21*_
*A2B2*	*x*_*22*_	*B* **2**	*q*_*2*_ *= x*_*12*_ *+ x*_*22*_

**Figure 1:**
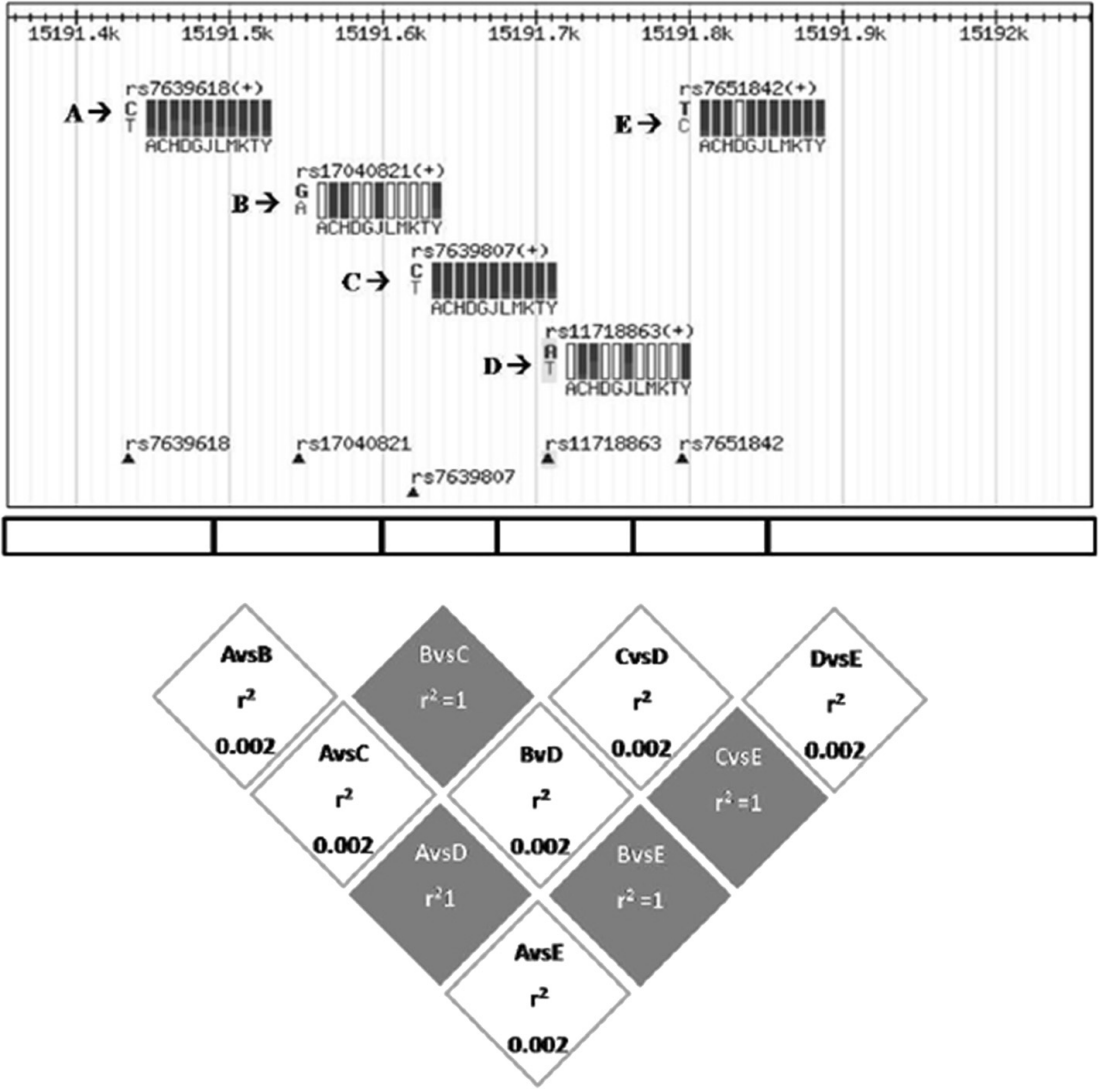
**This figure display the r2 LD plot, where the perfect LD between SNPs pair is (r2 = 1) dark marked, whereas the LD absence (r2 = 0) is white marked.**

Finally, similarly to the Haploview LD output form, we elaborated a r^2^ LD plot, where the perfect LD between SNPs pair is described by r^2^ = 1 and is dark marked, whereas the LD absence is described by r^2^ = 0 and is white marked (Figure 1).

### Mutational analysis of OA susceptibility genes

Genotyping analyses were performed by sequencing analysis of amplicons of 924 bp, containing the two DVWA SNPs: rs11718863, rs7639618. Sequencing analysis of the electropherograms revealed the presence of others three less known DVWA SNPs: rs7651842, rs7639807 and rs17040821. Sixty-one osteoarthritis patients and one hundred healthy subjects were genotyped for the above-mentioned five DVWA SNPs. Percentages of the wild type (WT), heterozygote (H) and homozygote (MUT) genotypes for each polymorphism were calculated. SNPs, genotype percentages and allele frequencies of 161 individuals investigated in this study are also reported (Table 2). For OA patients, we reported genotyping data of the three radiographic groups (A, B, C) and the number of individuals for each genotype. Deviations of Hardy-Weinberg equilibrium for all polymorphisms analyzed were not observed (Table 3).

**Table 2:** **Genetic analysis results**

**DVWA polymorhism**	**Genotype**	**OA patients n = 61**	**%**	**Allele frequencies**	**%**	**Controls n = 100**	**%**	**Allele frequencies**	**%**
rs11718863									
TT	WT	41	67	T = 100	82	72	72	T = 100	72
TA	H	18	30	A = 22	18	25	25	A = 56	28
AA	MUT	2	3			3	3		
rs7639618									
GG	WT	41	67	G = 100	82	72	72	G = 144	72
GA	H	18	30	A = 22	18	25	25	A = 56	28
AA	MUT	2	3			3	3		
rs7651842									
AA	WT	44	72,1	A = 103	84,4	85	85	A = 170	85
AG	H	15	24,6	G = 19	15,6	14	14	G = 30	15
GG	MUT	2	3,3			1	1		
rs7639807									
GG	WT	44	72,1	A = 103	84,4	85	85	G = 170	85
GA	H	15	24,6	G = 19	15,6	14	14	A = 30	15
AA	MUT	2	3,3			1	1		
rs17040821									
CC	WT	44	72,1	C = 103	84,4	85	85	C = 170	85
CT	H	15	24,6	T = 19	15,6	14	14	T = 30	15
TT	MUT	2	3,3			1	1		

n = number of patients.

**Table 3:** **Genetic analysis results according to KL grading groups**

					**KL grading groups**					
**DVWA polymorphism**	**Genotype**	**A group n = 20**	**%**	**HWE**	**B group n = 21**	**%**	**HWE**	**C group n = 20**	**%**	**HWE**
rs11718863										
TT	WT	15	75		17	81		9	45	
TA	H	4	20	0,33	4	19	0,63	10	50	0,39
AA	MUT	1	5		0	0		1	5	
rs7639618										
GG	WT	15	75		17	81		9	45	
GA	H	4	20	0,33	4	19	0,63	10	50	0,39
AA	MUT	1	5		0	0		1	5	
rs7651842										
AA	WT	14	70		15	71,4		15	75	
AG	H	6	30	0,43	5	23,8	0,51	4	20	0,33
GG	MUT	0			1	4,8		1	5	
rs7639807										
GG	WT	14	70		15	71,4		15	75	
GA	H	6	30	0,43	5	23,8	0,51	4	20	0,33
AA	MUT	0			1	4,8		1	5	
rs17040821										
CC	WT	14	70		15	71,4		15	75	
CT	H	6	30	0,43	5	23,8	0,51	4	20	0,33
TT	MUT	0			1	4,8		1	5	

### KL and genotype association analysis

To evaluate a potential association between genotypes, WT group or Mutated (Mut + H) one and the KL groups (A, B, C), Mann Whitney-U test, Chi-Square test and Fisher’s exact test were performed (Table 4). Analysis showed a statistically significant association between genotype and KL grading scale for the rs11718863 and rs7639618 DVWA polymorphisms (p = 0.03). These results are in line with the study of Valdes AM et al [21], where these polymorphisms are associated with the risk of knee OA in the UK population, but to our knowledge, this is the first study that reports the simultaneous presence of these two genetic alterations associated with KL in a Sicilian group. Finally, it is possible to note in Table 4, that rs11718863 and rs7639618 DVWA SNPs (genotype H + Mut) are more represented in group C (55%), compared to the other two groups A (25%) and B (19%), suggesting that they can be associated with a more severe OA radiographic grade.

**Table 4:** **Genotype and alleles statistical association with KL grade**

	**KL and genotype**						**KL and alleles**					
**DVWA polymorphism**		**WT**	**%**	**H/Mut**	**%**	p-value*		**Allele T**	**%**	**Allele A**	**%**	p-value*
	A	15	75	5	25		A	34	85	6	15	
rs11718863	B	17	81	4	19	0,03	B	38	90,5	4	9,5	0,04
	C	9	45	11	55		C	28	70	12	30	
								**Allele G**		**Allele A**		
	A	15	75	5	25		A	34	85	6	15	
rs7639618	B	17	81	4	19	0,03	B	38	90,5	4	9,5	0,04
	C	9	45	11	55		C	28	70	12	30	
								**Allele A**		**Allele G**		
	A	14	70	6	30		A	34	85	6	25	
rs7651842	B	15	71,4	6	28,6	0,936	B	35	83,3	7	16,7	0,9713
	C	15	75	5	25		C	34	85	6	25	
								**Allele G**		**Allele A**		
	A	14	70	6	30		A	34	85	6	25	
rs7639807	B	15	71,4	6	28,6	0,936	B	35	83,3	7	16,7	0,9713
	C	15	75	5	25		C	34	85	6	25	
								**Allele C**		**Allele T**		
	A	14	70	6	30		A	34	85	6	25	
rs17040821	B	15	71,4	6	28,6	0,936	B	35	83,3	7	16,7	0,9713
	C	15	75	5	25		C	34	85	6	25	

*Chi-Squared Test.

For rs7651842, rs7639807 and rs17040821 DVWA SNPs, we did not observed significant statistical association with radiographic KL grade (Table 4).

## Discussion

Nowadays, molecular genetic investigations have gained an increasingly significant role in the completion of OA etiology and provided evidence for a genetic contribute to OA. We previously reported a relationship between genetic, clinical and radiographic features in OA disease, and we suggested to evaluate OS5 and OS6 SNPs as biomarkers of OA grading and progression [20].

In this work, we recruited 61 OA Sicilian cases divided into 3 groups (A, B and C), based on degree of KL radiographic knee OA and 100 healthy individuals, from the same geographic area.

**Figure 2:**
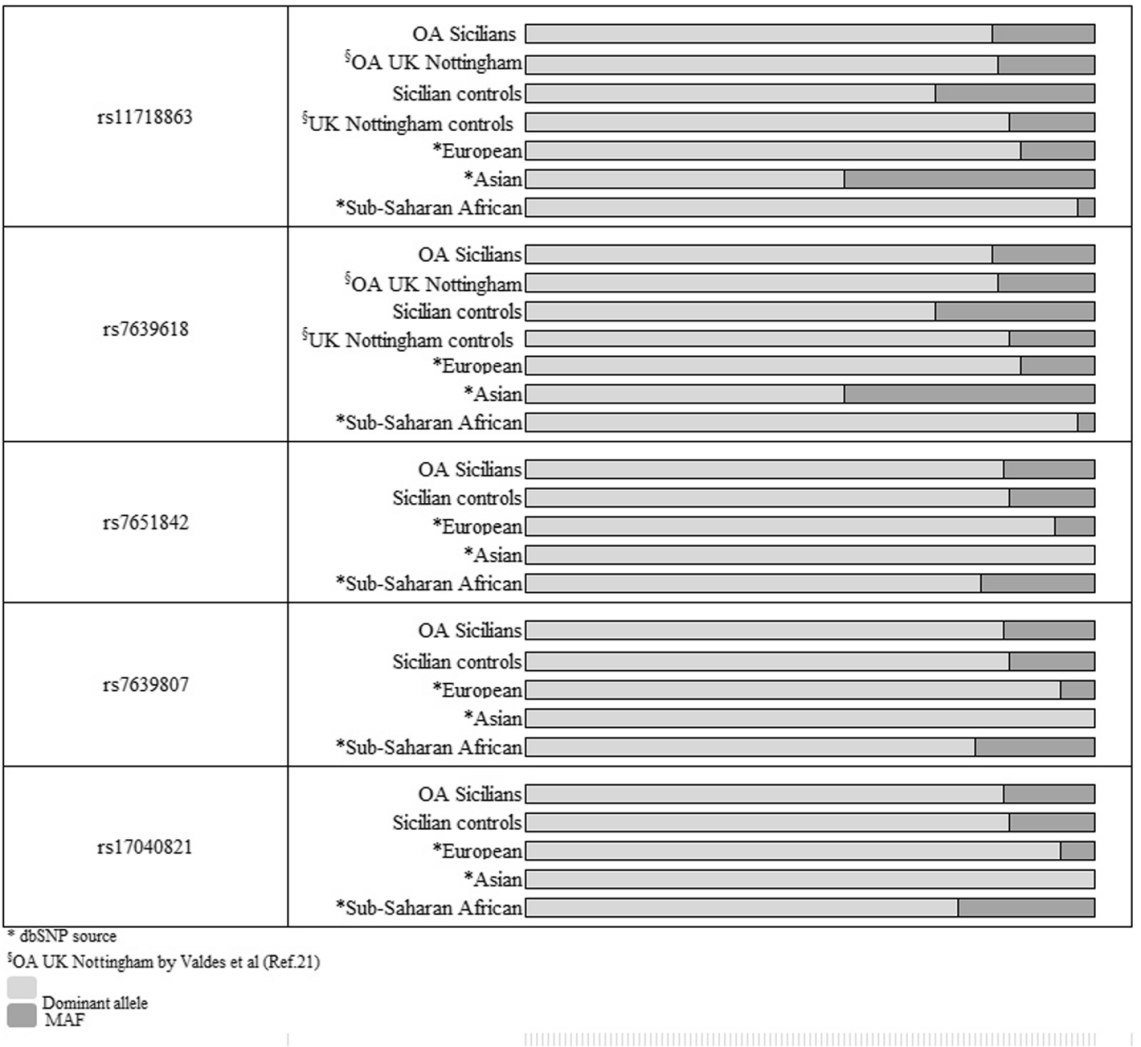
**SNPs alleles frequencies and MAF values for individuals of various geographic areas.**

Concerning the rs11718863 (OS6) and rs7639618 DVWA genetic polymorphisms, alleles frequencies analyzed in this work were different in the Sicilian group with respect to those reported in dbSNP database for European individuals of various geographic areas (Figure 2). In line with data obtained by Meulenbelt et al. and Valdes et al [21], the MAF for these SNPs were much lower in the European samples than in Asian ones. In addition, for the two above-mentioned genetic alterations, we have shown that MAF values were greater in the Sicilian individuals than in the European ones: 18% rather than 14.6% and 13%, according to data reported by Valdes and colleagues [21] and in SNPs database respectively [26].

It is well known that polymorphisms are involved in biodiversity and could be affected by ethnic heritage and geographic localization. In order to evaluate their hypothetical roles in human disease, such as OA, different geographical regions should be investigated to ascertain biodiversity and to fully understand the SNP variant effects.

Interestingly, a perfect genotypic correspondence (100% of the cases) has been displayed in all of the 161 individuals investigated, between rs11718863 and rs7639618 DVWA SNPs. In other words, all individuals are double WT or double H or double MUT carriers. In order to verify the degree of allelic segregation among these SNPs, we performed a Linkage Disequilibrium Analysis. The r^2^ test, according to the observation of a coinheritance of SNP alleles strongly suggests that rs11718863 and rs7639618 DVWA SNPs, segregate as haplotype (r^2^ = 1) (Figure 1), in line with data published by Miyamoto and colleagues, but for the first time described in Sicilian individuals analyzed in our study [29]. Considering that rs11718863 DVWA SNP is marked as susceptibility site, and that we have shown that it is in LD with rs7639618 DVWA, we suggest to assay also this genetic alteration in OA patients in order to define the functional role of DVWA in OA grading and progression.

In addition, even for rs7651842, rs7639807 and rs17040821, sequencing data analysis have displayed a perfect genotypic correspondence (100% of the cases) in all of the 161 individuals investigated. In other words, all individuals were triple WT or triple H or triple MUT carriers. Interestingly, three of the 161 individuals investigated were triple MUT, also taking into consideration that in the literature data these genotypes occur separately at low percentages (rs7651842: 0.9%; rs7639807: 1.7%; rs17040821:1.7%). Therefore, we calculated r^2^ LD coefficient, using a pairwise approach which resulted r^2^ = 1 in every SNP pair analyzed, suggesting that the above-mentioned SNPs segregate as haplotype (Figure 1). To our knowledge, these assumptions have been described for the first time in a cohort of Sicilian individuals, even if a not perfect haplotype segregation is expected, considering the MAFs reported in dbSNP database. Moreover, the observation that no MAF alleles are detected in dbSNP database for the Asian population, supports the need of additional studies on Caucasian or Sicilian individuals to better investigate the role of rs7651842, rs7639807 and rs17040821 SNPs and their possible contribution on DVWA protein functional changes and their impact on OA susceptibility in people of other geographic areas.

We recently reported that rs11718863 was statistically associated with KL values in a cohort of Sicilian OA patients and also that this genetic alteration was more represented in individuals with more severe knee OA KL radiographic grading. In order to extend the association with KL grade also for the rs7639618 DVWA SNP, we tested the significance of rs7639618 allele dose distribution among the KL OA groups showing a significant association between rs7639618 and KL in our OA patients cohort.

Sequencing analysis of the electropherograms revealed the presence of other three less known DVWA SNPs: rs7651842, rs7639807 and rs17040821, for which no literature data were available. Also for these SNPs, MAF values were approximately three fold greater in the Sicilian individuals than in the European ones (Figure 2).

Unlike rs11718863 and rs7639618, these polymorphisms are not marked as *clinically relevant* in dbSNP database probably due to absence of bibliographic data. These three genetic alterations are located in the same exonic region of DVWA gene and for this reason we hypothesize that they could cause protein functional changes, not still investigated.

Finally, in our study a high percentage of the 161 Sicilian individuals are carriers of DVWA SNPs mutated alleles. In particular, 29.8% were H or homozygous MUT for rs11718863 (OS6) and rs7639618, whereas, 19.9% were H or homozygous MUT for rs7651842, 7639807 and rs17040821 SNPs.

A limitation of this study may be our small sample size. This may influence the generalizability of these findings; however, our results are representative of patients with mild to moderate knee OA. In addition, the positive association described in this paper could represent a preliminary study that highlights DVWA gene role in OA patients and in particular, for Sicilian individuals.

Summing up, DVWA gene was recently related to OA through a genome wide association study in the Japanese population. Today, a very limited number of reports have clarified its molecular role on cartilage homeostasis, even if some authors and association studies have supposed its role in OA etiopathogenesis [4,26,27,28,29,31-40]. Additional studies are needed to clarify the SNPs contribution to functional protein modifications, also to highlight which SNPs are more important on OA susceptibility in different geographic areas.

## Conclusion

Highlighting genetic basis of human disease could contribute to improve personalized treatments [44,45]. OA is the most common joint disorder affecting large segments of the population and leading to significant disability and impaired quality of life. The purpose of this preliminary research was to highlight possible associations between DVWA SNPs and OA clinical and radiographic data. There are several lines of evidence indicating that genetic abnormalities can result in early onset of OA. In this work we genotyped 61 OA patients and 100 control samples. For all the DVWA SNPs investigated, the MAF values were higher in our Sicilian cohort than in the European one. Additional studies are needed in order to clarify these SNPs distribution in people of different geographic areas. As previously described for rs11718863 SNP, also the rs7639618 polymorphism has shown a statistical association with KL grade, but not observed for the rs7651842, rs7639807 and rs17040821. Thus, we suggest to test also the rs7639618 SNP in OA patients. This work represents a multidisciplinary and translational medicine approach to study OA where clinical, radiographic and genetic evaluation could contribute to better define the disease grading and progression for the development of new therapies.
